# Advancing reliability and efficiency of urban communication: Unmanned aerial vehicles, intelligent reflection surfaces, and deep learning techniques

**DOI:** 10.1016/j.heliyon.2024.e32472

**Published:** 2024-06-05

**Authors:** Chongyang Li, Xiaohu Qiang

**Affiliations:** Hunan Post And Telecommunication College, Hunan Changsha, 410015, China

**Keywords:** Unmanned aerial vehicles (UAVs), Channel modeling, Artificial intelligent (AI), Intelligent reflection surfaces (IRS), Deep learning (DL)

## Abstract

Unmanned aerial vehicles (UAVs) have garnered attention for their potential to improve wireless communication networks by establishing line-of-sight (LoS) connections. However, urban environments pose challenges such as tall buildings and trees, impacting communication pathways. Intelligent reflection surfaces (IRSs) offer a solution by creating virtual LoS routes through signal reflection, enhancing reliability and coverage. This paper presents a three-dimensional dynamic channel model for UAV-assisted communication systems with IRSs. Additionally, it proposes a novel channel-tracking approach using deep learning and artificial intelligence techniques, comprising preliminary estimation with a deep neural network and continuous monitoring with a Stacked Bidirectional Long and Short-Term Memory (Bi-LSTM) model. Simulation results demonstrate faster convergence and superior performance compared to benchmarks, highlighting the effectiveness of integrating IRSs into UAV-enabled communication for enhanced reliability and efficiency.

## 1 Introduction

Unmanned aerial vehicles, commonly referred to as UAVs, hold promise as aerial base stations, offering benefits such as extended coverage, increased connection reliability, and reduced energy consumption in communication systems [1]. The adaptability of Unmanned Aerial Systems (UASs) has led to a wide range of potential applications, including security surveillance, real-time monitoring, search and rescue missions, and emergency communication [2]. Despite the promising potential of AI-assisted systems for future wireless communications in smart urban environments, the complex urban landscape presents challenges regarding Line-of-Sight (LoS) networks between navigating AIs and terrestrial users. In this context, Intelligent Reflection Surfaces (IRSs) have emerged as crucial tools for overcoming signal attenuation and strengthening communication lines by establishing virtual LoS pathways [3]. The integration of IRSs aims to enable cost-effective modulation of phase shifts, strategically directing signal strength to mitigate information loss. Thus, the integration of IRSs serves as a viable solution to address the obstacles faced by AI-assisted systems [4], extending pervasive communication services facilitated by AI and alleviating challenges posed by distant users in relation to AI navigation.

The synergy between IRSs and artificial intelligence (AIs) within the wireless ecosystem promises significant enhancements in communication performance, driven by their inherent advantages. Moreover, precise channel state information (CSI) acquisition is essential for optimizing transmissions to ensure superior communication quality. However, tracking channels in IRS-supported AI systems presents a more complex challenge compared to CSIestimation in traditional communication setups due to factors such as the extensive array of reflecting elements within the IRS, the mobility of both AIs and human entities, and limitations in signal processing capabilities.

Despite the potential of LoS connectivity provided by UAVs to establish reliable communication channels and reduce communication distances, obstacles such as natural barriers like trees and tall buildings may occasionally disrupt communication pathways. In the realm of innovative communication paradigms, the integration of IRSs into AI communication methodologies has garnered increasing scholarly interest [5]. For example, previous research explored the combination of IRS beamforming and AI trajectory optimization to enhance the mean achievable data rate of the system. Additionally, investigations into the energetics of using an IRS to empower a mobile AI relay have highlighted the intricate relationship between energy efficiency and communication efficacy [6].

Research examining UAV relaying systems augmented by IR) positioned within buildings, aimed at facilitating communication between the device and AI, has been meticulously scrutinized. Similarly, studies involving cooperative passive beamforming of the IRS alongside AI position optimization have been conducted to reduce error rates during data decoding operations [7]. It is important to note that these studies presume foreknowledge of CSI, emphasizing the need for robust channel-tracking mechanisms tailored to IRS-empowered AI communication systems. Factors such as robust estimation performance, efficient pilot overhead management, and the impact of time-varying channels resulting from the mobility of communicating entities underscore the urgency of developing such mechanisms. A comprehensive survey of existing literature has spurred the development of a Deep Learning (DL)-based channel locating algorithm calibrated for IRS-assisted systems, promising not only a reduction in training costs but also an enhancement in tracking precision.

The ensuing enumeration encapsulates the pivotal contributions borne by this study.

1. To the best of our knowledge, the present endeavor stands as the maiden effort to formulate a dynamic channel model grounded in 3D geometry for IRS-enhanced communication systems integrated with UAVs, catering to both stationary IRS configurations, navigating AIs, and mobile user units. The methodological pursuit is characterized by the derivation of a time-varying channel model achieved through the amalgamation of a dynamic cascade of virtual LoS connections (user-IRS-AI) underscored by an activation parameter, in conjunction with a dynamic LoS link (user-AI) articulated by a blockage parameter. Within the construct of the system model, a comprehensive delineation encompasses parameters encompassing velocities, maximal Doppler effects, propagation delays, and temporal delays, elucidating the intricate facets that collectively mold the operational fabric of the proposed system.
2. Within the configured architectural paradigm, a channel tracking apparatus grounded in DL principles is propounded to orchestrate the continual monitoring of the temporally evolving communication channel. The delineation of the proposed mechanism discernibly bifurcates into two salient components. The pre-estimation facet is effectuated through the offline training regimen of a deep neural network (DNN) fortified with a repository of meticulously amassed training datasets. On the other front, the tracking module integrates the innovative Bi-directional Long Short-Term Memory, a data-driven architecture adept at adeptly capturing the evolution of CSI within a dynamic channel milieu. This diligently orchestrated, characterized by a bidirectional configuration spanning multiple layers aggregated with a historically informed trace-back phase within the chronological time sequence.
3. In juxtaposition with established benchmark methodologies, the advanced channel tracking algorithm articulated in this study exhibits a propensity for achieving convergence of the loss function within a reduced number of offline training epochs. Furthermore, computational simulations duly manifest that the novel approach, epitomized by the tandem interplay of ‘DNN followed by the Bi-directional Long and ‘Short-Term Memory (Stacked Bi-LSTM), mirrors complexity profiles inherent in the benchmark algorithms. However, surpassing them in both the realm of channel tracking efficacy and the management of pilot overheads stands as the hallmark of its superior performance.

## 2 Literature review

An innovative approach to college English education involves the integration of virtual reality (VR), artificial intelligence (AI), and machine learning technologies. This integrated approach aims to enhance experiential learning by creating immersive and interactive environments tailored to various learning styles, thus fostering better English language acquisition. Utilizing virtual reality (VR) and AI technologies empowers educators to construct dynamic learning environments that cater to diverse student needs. AI-powered speech recognition systems play a crucial role in improving listening and oral communication skills, thereby enhancing classroom instruction effectiveness. Moreover, the fusion of deep learning and VR presents a novel method for English education, stimulating deep learning and student engagement through innovative teaching methodologies. The integration of these advanced technologies has the potential to revolutionize college English teaching, making it more engaging, effective, and adaptable to the challenges posed by the Fourth Industrial Revolution and digital transformation.

Upcoming wireless communication paradigms need to confront the extensively explored challenge of realizing wireless communication that is both ultra-reliable and high-capacity within dynamic environments. Implementing power and beam forming control, as well as developing efficient coding and modulation algorithms, have all received a lot of attention. However, the wireless connection that connects the communication endpoints is ignored in these analyses. Recent years have seen a lot of interest in emerging technologies, including communications made possible by AIs and IRS [8]. AI-based aerial base stations have a height and mobility advantage over terrestrial base stations. AIs are particularly beneficial in wireless communications because of their versatility. AIs in particular can support energy-constrained and short-range communication equipment such as sensors and monitoring [9]. In spite of their applicability across a wide spectrum of communication scenarios, the currently employed statistical (MIMO) channel models are not capable enough in their ability capturing distinct characteristics, such as the three-dimensional (3D) spatial mobility exhibited by AIs at elevated altitudes [10]. Recent investigations [11] have introduced geometry-based channel models tailored to AIs for air-to-ground communications. This study marked the pioneering attempt to comprehensively assess AI mobility spanning velocity vectors and directional changes within the plane, as documented in Ref. [12]. Additionally, the papers in deduced time-varying parameters referring to angles of ‘arrival (AoAs) and ‘departure (AoDs) to effectively represent the channel's dynamic non-stationarity due to AI movement. Notably [13], focused solely on a single non-Line-of-Sight link and overlooked the mobility of scatterers. On a related note, delved into cluster-based propagation channels, while [14] extended the investigation to include initial azimuth, elevation, angle of arrival (AoA), and angle of departure (AoD) considerations.

Alongside the potential of AI-assisted communications to offer efficient connectivity, the incorporation of IRSs into wireless communication systems has garnered considerable attention. The utilization of IRSs can extend to various applications including road signs, architectural exteriors, ceilings, indoor walls, and even portable devices worn by pedestrians [15]. It is composed of a variety of low-cost and energy-efficient reflecting pieces. By altering the phase shifts of the IRS elements, it becomes possible to disperse the reflected signals, thereby enabling the creation of simulated LoS connections. This results in better signal strength [16]. A system with IRSsupport also enhances spectral efficiency and communication coverage [17]. The prospective efficacy of communication facilitated by the IRS critically relies upon its adeptness in capturing and comprehending intricate CSI. Consequently, the practical deployment of IRS encounters a formidable challenge, necessitating the deployment of sophisticated less complex tracking strategies [18]. The contemporary research landscape spans the spectrum from discussed to specific communication scenarios, all targeted at channel estimation within IRS-augmented systems. Addressing the realm of conventional IRS-assisted Multi-User (MU) communications, an innovative three-phase pilot-based channel estimation paradigm was introduced by Ref. [19]. This novel approach entails the meticulous dissection of individual links during distinct phases [20]. These sequential and simultaneous user channel estimation techniques used operate with acute consideration for the salient links within authentic real-world environments. Moreover, delved into scenarios while considering the partial activation of IRS's reflective constituents. For scenarios that deviate from the conventional, such as confined indoor domains characterized by obstructed LoS paths [21] unveiled an innovative IRS-assisted channel model. This conceptual framework assigns primacy to the virtual LoS path, engendered by IRS reflection, over the physical LoS path. Pushing the envelope further [22], ventured into the domain of double-IRS cooperatively augmented Multiple-Input Multiple-Output (MIMO) systems, encompassing a cascade of single-reflection and double-reflection links. The ascent of Deep Learning (DL) methodologies, with their inherent capacity to transcend linear correlations, has imparted a revolutionary contour to communication systems. These methodologies, poised to supplant conventional model-driven channel estimation paradigms, have made significant inroads. Delineating a profound departure from established norms, presented a ‘deep learning paradigm for ‘estimation’ in multi-user featuring IRS, encompassing both physical and virtual LoS connections. Similarly, propounded a learned approximation pass message network tailored to the intricacies of beam space millimeter-wave large MIMO systems. Echoing this theme [23] unfolded a novel approach predicated on deep denoising neural networks, orchestrating a virtuous interplay with Compressed Sensing (CS) channel estimation strategies within IRS systems [24]. in a parallel vein, engineered a DL-centric approach that endowed IRS with an acumen to judiciously interface with signals. Pertinently, engineered a dual Convolutional Neural Network (CNN) architecture as the keystone for IRS-driven MIMO systems, orchestrating estimations for both LoS and cascaded non-LoS propagation paths. The confluence of these endeavours hinges on the tacit supposition of a static environment and immobile mobile users, a premise that aligns with channel estimation in IRS-facilitated communication systems. Yet, the actual operational landscape scarcely aligns with this convenient assumption, necessitating an astute deliberation on the intricate contours of time-varying channel estimation or tracking. Notably, the DL-based channel estimation methodologies proffered in are predominantly underpinned by offline training paradigms. Curiously, none of the extant studies traverse the avenue of harnessing historical data's chronology to effectuate a nuanced trajectory of channel monitoring over time.

## 3 Method and data

We employ the following standard notation throughout the paper. The letter ℂ stands for complex numbers. The notations employed for the transpose and for the second that is a conjugate transpose operation is represented as ${\;\left(\cdot \right)}^{T}$, and ${\left(\cdot \right)}^{H}$. The identity matrix is symbolized as I. A complex Gaussian random vector, characterized by a predetermined mean and covariance structure, is denoted utilizing the nomenclature CN (Mean, covariance). The matrix trace is denoted by the symbol tr(·). The Kronecker product is shown as A⊗B. The direct sum is indicated by A⊕B. The $l_{2}$ norm is used to represent expectation as ‖.‖.

The focus of this research encompasses an uplink scenario involving a multi-user system empowered by an IRS in conjunction with UAVs. This configuration is characterized by an M element antenna array integrated onto the AI, alongside N reflective elements strategically positioned upon the IRS, catering to the communication needs of K users. For this analytical framework, each individual $k^{th}$ user is presumed integration with a solitary antenna module. Core impetus underlying this approach is the augmentation of the wireless communication quality that underpins interactions between the AI and the mobile user cohort. To this end, the IRS is strategically deployed atop a high-rise edifice, thereby engendering extended coverage and concurrently curbing the necessity for extensive AI mobility, as evidenced by Ref. [25].

**Fig. 1:**
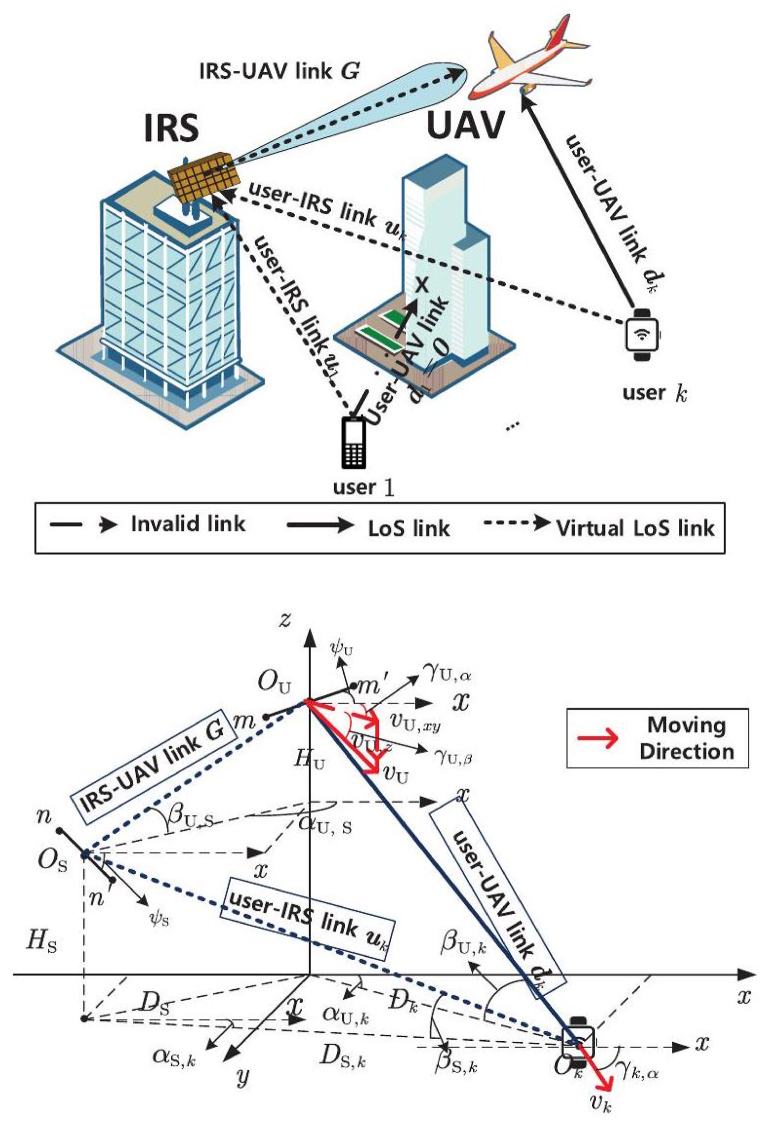
The diagram depicts a multi-user communication system facilitated by Unmanned Aerial Vehicles (UAVs) with assistance from the Intelligent Reflecting Surface (IRS). The present study focuses on the system model pertaining to the uplink of multi-user communication facilitated by unmanned aerial vehicles (UAVs) with the assistance of intelligent reflecting surfaces (IRS).

Illustrated in Fig. 1 (a), the system architecture is characterized by two principal link categories: The Line-of-Sight h(LoS) link connecting the user and the AI, and the virtual LoSi link interlinking the user, IRS, and AI. The $k^{th}$ that is a user-AIlink existing at an instance t is mathematically represented as ${\mathrm{d}}_{k}\left(t\right)\in C^{M\times 1}$. In parallel, the $k^{th}$ (user) ‘IRS-AI virtual LoS link, also evolving at time t, encompasses distinct LoS sub-links:having the user-IRSu sub-link $u_{k}\left(t\right)\in C^{M\times 1}$, and the “IRS-AI” sub-link $\left(t\right)\in C^{M\times N}$. Detailed exposition regarding the dynamic intricacies of the 3D IRS-assisted having a channel model is meticulously presented in the visual depiction encapsulated within Fig. 1(b). The velocities characterizing the Unmanned Aerial Vehicle (UAV) and the $k^{th}$ user are denoted as $v_{U}$ and $v_{k}$, respectively.

The basic is the LoS connection prevailing between $k^{th}$ and the AI during time instance t is encapsulated ${\mathrm{d}}_{k}\left(t\right)={\left[{\mathrm{d}}_{k,m}\;\left(t,\tau \;\right)\right]}_{M\times 1}$ in Eq (1).

(1) $$\begin{array}{c} {\mathrm{d}}_{k,m}\;\left(t,\tau \;\right)=\Omega_{K,m}e-j2\pi fc\tau k,m\;\delta \left(\tau \;-\;\tau_{U,k,LoS}\right)\\ \begin{array}{c} \times {\mathrm{e}}^{j2\pi fkt\left[\cos\left(\alpha U,k-\gamma k,\alpha \right)\cos\;\beta U,kcos\gamma k,\beta +\sin\;\beta U,ksin\gamma k,\beta \;\right]}\\ \times {\mathrm{e}}^{j2\pi fUt\left[\cos\left(\alpha U,k-\gamma U,\alpha \right)\cos\;\beta U,k\right]} \end{array} \end{array}$$

In this context, $\Omega_{K,m}$ signifies the attenuation factor characterizing the link between the $m^{th}{\left(0\leq m^{\prime }\leq m^{\prime }\times \leq M\right)}^{2}$ antenna elemention the AI and the single antenna positioned on the ${\;k}^{th}$ user. The parameter $f_{c}$ corresponds to the carrier frequency, while $\tau_{k,m}=L_{k,m\;}/c$ stands as the lag of waves traversing in the ${\;m}^{th}$ antenna element on the AI and the single antenna on the ${\;k}^{th}$ at the specific instance *t*, with $L_{k,m\;}$ representing the distance separating the user and the ${\;m}^{th}$ antenna element. $\tau_{U,k,LoS}$ encapsulates the temporal delay intrinsic to the ${\;k}^{th}$ user-AI LoS link, operative during time *t*. Additionally, $f_{U}=\frac{v_{u}}{\lambda }\;and\;f_{k}=\frac{u_{k}}{\lambda }$ signify the maximal Doppler frequency, stemming from the motion of both the AI and the ${\;k}^{th}$ user, wherein λ represents the wavelength. Further attributes include $\alpha_{U,k,}$ and $\beta_{U,k}$ which denote the azimuth and elevation angles corresponding to the orientation between the ${\;k}^{th}$ user and the AI. Likewise, $\gamma_{U,\alpha ,}$ and $\gamma_{U,\beta }$ designate the azimuth and elevation angles characterizing the trajectory of AI's motion. Furthermore, $\gamma_{k,\alpha }$ pertains to the azimuth angle delineating the direction of motion for the k'th user, relative to the AI's location. This complex interplay culminates in the time delay inherent to this LoS connection Eq (2).

(2) $$\tau U,k,LoS=\frac{D_{K}}{c0\;\cos\;\beta U,k}$$

Having $D_{k}$ denote the *xy*-plane antenna center distance separating the AI and the ${\;k}^{th}$ user, while $C_{0}$ represents the speed of light, it's noteworthy to emphasize that, when contrasting the virtual LoS connection to each of the system sub-links delineated by $d_{k,m}\left(t,\tau \;\right)$ in (1), the principal distinction stems from the fixed placement of the Intelligent Reflection Surface (IRS) without any movement, effectively denoting $f_{S}=0$. Therefore, the ${\;k}^{th}$ user-IRS LoS sub-link, operational during that instance t, assumes the configuration $u_{k}{\left(t\right)=[u}_{k,n}{\left(t,\tau \;\right)]}_{N\times 1}$, with N representing the number of elements in the sub-link in Eq (3).

(3) uk,n(t,τ)=Ωk,ne−j2πfcτk,nδ(τ−τS,k,LoS)×ej2πfkt[cos(αS,k−γk,α)cosβS,k]

The inclusion of the ${\;m}^{\prime th}$ element within the illustrative depiction, as showcased in Fig. 1, serves the purpose of elucidating the antenna's planar orientation angle, denoted as ψU. In a manner analogous to the presence of the ${\;n}^{\prime th}$ reflectionk element on the IRS, the tangible flat angle finds its expression through the notation ψS.

The attenuation factor see Table 1, designated as Ω(k,n), embodies the quantification of signal attenuation transpiring between the ${\;n}^{th}\left(0\leq n\leq n^{\prime }\leq N\right)$ reflecting element on the IRS and the individual antenna affixed to the ${\;k}^{th}$ user. The index n spans the range from 0 to n × , where n × is bounded by N. The variable τS,k,LoS encapsulates the temporal lag within the LoS sub-link interlinking the ${\;k}^{th}$ user and the IRS during the instant t. Furthermore, the parameters α(S,k) and β(S,k) assume significance as they delineate the azimuth and elevation orientations, respectively, underscoring the angular interplay between the ${\;k}^{th}$ operator and the IRS system. Meanwhile, γk,α designates the orientation of the azimuth angle encompassing the motion direction of the ${\;k}^{th}$ user. To elucidate the time delay associated with wave propagation spanning the ${\;n}^{th}$ to that antenna element on the IRS and the solitary antenna unit stationed upon the ${\;k}^{th}$ user at time t, the parameter τk,n is invoked. Within this context, $L_{k,n\;}$ conveys spatial separation between the user and the ${\;n}^{th}$ element situated on the IRS in Eq (4). The physical constant, embodying the speed of light, is succinctly denoted as C.

(4) $$u_{k,n}\;\left(t,\tau \;\right)=\Omega k,n{\mathrm{e}}^{-j2\pi fc\tau k,n}\;\delta \;\left(\tau \;-\;\tau S,k,LoS\right)\times {\mathrm{e}}^{j2\pi fkt\left[\cos\left(\alpha S,k-\gamma k,\alpha \right)\cos\;\beta S,k\right]}$$

**Table 1:** Table of significate symbols.

Symbols	Explanations
$M,m,m^{\prime }$	Aggregate count and sequential designation of AI's antenna constituents
$N,n,n^{\prime }$	Cumulative count and sequential labeling of components comprising the Intelligent Reflecting Surface (IRS)
$N_{p}$	The overall quantity of a training pilot.
$\alpha_{U,k},\beta_{U,k\;}$	The angular positions denoting the horizontal (azimuth) and vertical (elevation) orientations of both arrival and departure (AoAs/AoDs) in the communication link between the user and the unmanned aerial vehicle (UAV).
$\alpha_{S,k},\beta_{S,k\;}$	The angular positions denoting the horizontal (azimuth) and vertical (elevation) orientations of both arrival and departure (AoAs/AoDs) in the user-intelligent Reflecting Surface (IRS) connection.
$\alpha_{U,S},\beta_{U,S\;}$	The angular positions denoting the horizontal (azimuth) and vertical (elevation) orientations of both arrival and departure (AoAs/AoDs) in the context of the connection between both systems.
$r_{U,\alpha },r_{U,\beta \;}$	The azimuth and elevation angles of unmanned aerial vehicle (AI) movement.
$r_{U,\alpha \;}$	The angular measurement of the $k^{th}$ user's movement.
$\beta_{T,n\;}$	The amplitude of the $n^{th}$ element of the Intelligent Reflecting Surface (IRS) at time slot t.
$\varphi_{t,n}$	The phase shift occurs at the $n^{th}$ element of the Intelligent Reflecting Surface (IRS) algorithm during time slot t.
$v_{U}$	AI movement speed
$v_{U,x,y},v_{U,z}$	The horizontal and perpendicular components of AI speed.
$v_{k}$	Speed of $k^{th}$ user movement
$f_{U}$	The maximum Doppler frequency of an Unmanned Aerial Vehicle (UAVs).
$f_{k}$	The maximum Doppler frequency of the $k^{th}$ user
$\psi_{U}$	The topic of interest is to the physical flat angle of antennas on unmanned aerial vehicles (UAVs).
$\psi_{S}$	The topic of interest is the physical characteristics of flat angles in the context of the IRS
$T_{k,m}$	The time lag associated with the transmission of a wave between the $m^{th}$ antennak element on the AI and the individual antenna of the $k^{th}$ user during time instance *t*.
$T_{k,n}$	The time lag associated with the transmission of a wave between the $n^{th}$ antenna element on the AI and the individual antenna of the $k^{th}$ user during time instance *t*.
$T_{m,n}$	The time lag associated with the transmission of the wave in AI $m^{th}$ antenna part element and $n^{th}$ users’ single antennas at time t
TU,k,LoS	Temporal lag of the link between the $k^{th}$ (user) and AI during time *t*
TS,k,LoS	Temporal lag of the link between the $k^{th}$ (users) and ‘IRS system LoS during time *t*
TU,S,LoS	Temporal lag of the link between the $k^{th}$ user, IRS-AI LoS during time *t*
$D_{k}$	The dimensional separation in the “XY-plane” between the AI and the $k^{th}$ user
$D_{S}$	The dimensional separation in the “XY-plane” between the AI and the IRS
$D_{S,k}$	The dimensional separation in the “XY-plane” between the (IRS) and the $k^{th}$ user
$\Omega_{k,m}$	The attenuation coefficient connecting the $m^{th}$ on the AI and the individual antenna of the $k^{th}$ user.
$\Omega_{k,n}$	The attenuation coefficient connecting the $n^{th}$ on the IRS and the individual antenna of the $k^{th}$ user.
$\Omega_{m,n}$	The attenuation coefficient connecting the AI $m^{th}$ (antenna element) and the IRS $n^{th}$ reflectionu element
$L_{k,m}$	The spatial separation between the $m^{th}$ (antenna element) on the AI and the singular kind of antenna of the $k^{th}$ users
$L_{k,n}$	The spatial separation between the $n^{th}$ (antenna element) on the IRS and the singular kind of antenna of the $k^{th}$ user.
$L_{m,n}$	The spatial separation the AI $m^{th}$ (antennas element) and the IRS $n^{th}$ reflection of element.

‘Sub-link at the time *t* on the IRS-AI *is* $G_{k}{\left(t\right)=[g}_{m,n}{\left(t,\tau \;\right)]}_{MN}$ with Eq 5

(5) $$gm,n\;\left(t,\tau \;\right)=\Omega_{m,n\;}{\mathrm{e}}^{\;j2\pi fc\tau m,n}\;\delta \left(\tau \;-\;\tau U,S,LoS\right)\times {\mathrm{e}}^{\;j2\pi fUt\left[\cos\left(\alpha U,S-\gamma U,\alpha \right)\cos\;\beta U,S\;\cos\;\gamma U,\beta +\sin\;\beta U,Ssin\gamma U,\beta \;\right]}$$

Within this framework, Ω(m,n) signifies the attenuation coefficient characterizing the link between the $m^{th}$ antenna element on the UAV and the $n^{th}$ element on the IRS. Meanwhile, τU,S,LoS is the parameter encapsulating the temporal lag intrinsic to the LoS’ sub-link interconnecting the IRS and AI at a given time instance *t*. Furthermore, α(U,S) and β(U,S) embody the angular azimuthj and increment orientations respectively, describing the spatial relationship between the AI and IRS. On a parallel note, τm,n represents the temporal delay attributed to wave propagation between the $m^{th}$ on the AI and the $n^{th}$ element on the IRS. Here, L(m,n) reduces the spatial separation amid these components. The constant c0 symbolizes the speed of light. The temporal lag inherent to two distinct sub-links during time t is delineated as follows Eq (6):

(6) $$\tau U,k,LoS=\frac{D_{S,K}}{c0\;\cos\;\beta S,k}$$

and Eq 7

(7) τU,S,LoS=DS/(c0cosβU,S),

The virtual LoS linking the $k^{th}$ user, IRS, and UAV at time *t* could be represented as $G\left(t\right)\Phi \left(t\right)u_{k}\left(t\right)\in CM\times 1$. In this equation, Φ(t)=diag(φ(t))∈CN×N stands for the “Phase shift matrix” attributed to the IRS, with $\varphi \left(t\right)={\left[{\beta t,1ej\varphi }^{t,1},\cdots ,{\beta t,nej\varphi }^{t,n},\cdots ,{\beta t,Nej\varphi }^{t,N}\right]}^{T}\epsilon \;C^{N\times 1}$, where $\beta_{t,n\;}\in [0\text{,}\;1]\;and\;\varphi_{t,n}\in [0\text{,}\;2\pi ]$. The investigation into the reflection amplitude and phase shift of IRS's subsurface component at time slot t has been explored.

Consequently, the AI-assisted uplink with IRS at time *t* can be represented as the amalgamation of the virtual LoS connection and the LoS link. This is succinctly expressed as follows in Eq 8.

**Figure:**
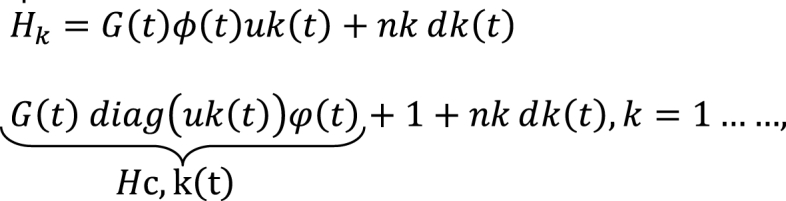

The cascaded channel between the kthj user, IRS, and AI during time slot t, denoted as HC,k(t)∈CM×N, is governed by the blockage parameter η∈{0,1}M×1. This parameter follows a Bernoulli distribution [26], wherein the blockage probabilities for the LoS connection are represented as pk.

The signal received by the UAV from the K user during interval slot t can be mathematically articulated as in Eq 9.

**Figure:**
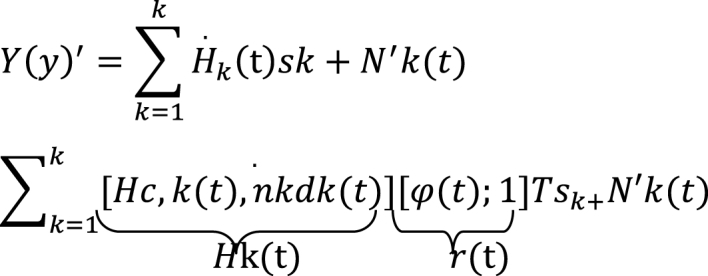

The signal $Y{\left(t\right)}^{\prime }\in CM^{\prime }\times Np$ symbolizes MU signal, while ${Hk\left(t\right))}^{\prime }\in CM\times \left(N+1\right)$ represents the channel matrix demanding estimation. In parallel, r(t)∈C(N+1)×1 embodies a vector, and sk∈C1×Np constitutes the pilot with its value being $8k8_{k}^{H}$. The equation $H=PNp\;8k_{a}\;8k_{b}=0$ stablishes a mathematical correlation, where p shows the power of user, and $k_{a},\text{and}\;k_{b}$ are integers spanning from 1toK, with the proviso that $k_{a}$ is not equivalent to $k_{b}$. The matrix $k_{a}\neq k_{b.}N^{\prime }b\left(t\right)∼CN\left(0,\sigma^{2}N,I\right)$ stands for the Additive White Gaussian Noise (AWGN) integrated within the system.

In scenarios involving multiple users, it is evident that the pilot sequences of any two users exhibit orthogonally. As a result, the received signal vectors pertaining to the $k_{th}$ user can be effectively disentangled by conducting a multiplication operation using the conjugate transpose of the sequence ${\;s\;}_{k}^{H}$ in Eq 10.

**Figure:**
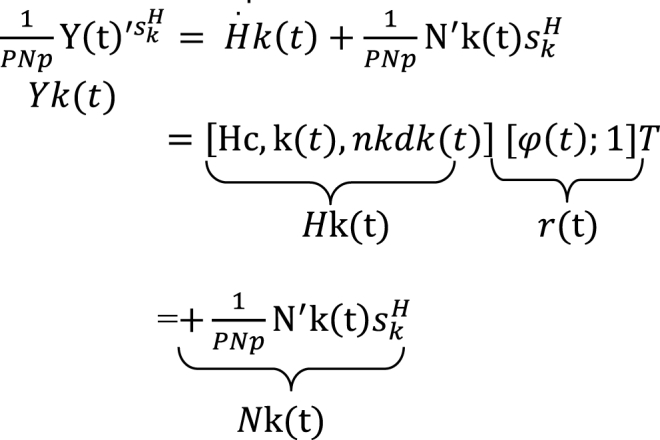

The signal vector received by the AI from the $k_{th}$ user is indicated as $k{\left(t\right)\in C}^{M\times Np}$^.^ The channel matrix necessitating estimation is denoted as $H_{k}{\left(t\right)\in C}^{M\times \left(N+1\right)}$. The noise component is expressed as r(t)∈C(N+1)×1, while the random variable $N_{k}\left(t\right)$ follows a complex Gaussian distribution with a mean of zero and a variance of ${(0,\sigma }^{2}\;N\;I)$.

The estimation of the channel $H_{k}\left(t\right)$ is performed using a channel tracker, represented as F(·), which relies on the received signal ${{\hat{Y}}^{\prime }\;\left(t\right),r\left(t\right)Y\;s}_{k}$. The channel ${\tilde{H}}_{k}\left(t\right)$ is approximated as a function F(Yk(t);r(t),sk). Therefore, the issue of pilot-aided channel estimation may be formulated as in Eq (11).

(11) $$\begin{array}{c} \mathrm{Min}\;E\;\left[\left\|Hk\left(t\right)-H_{k}\left(t\right)\right\|\right]\text{,}\\ s.t\;tr\;\left(sks_{k}^{H}\right)=Es\text{,} \end{array}$$

The energy limitation is represented as ${\;E}_{s}=N_{p}T_{s}P$, where Np represents the length of pilot overheads, $T_{s}$ defines the duration of a single time slot, and P indicates the transmit power.

In order to attract attention, the dynamic nature of both the UAV and the user from the (KTH) results in continuous fluctuations in the relative azimuth and elevation angles, as well as the relative distance, over time. The assumed starting location of the UAV is denoted as $\left(x_{U},y_{U},z_{U}\right)=\left(0,0,H_{U}\right)$. The $k_{th}$ user's position is represented as ($x_{k},y_{k},z_{k}$) = $\left(D_{k}\;\cos\;\alpha_{U,k},D_{k}\;\mathrm{Sin}\;\alpha_{U,k},0\right)$, where $D_{k}$ is the distance and $\alpha_{U,k}$ is the angle with respect to the AI. The position of the IRS is given as $\left(x_{S},y_{S},z_{S}\right)$ = ($D_{S}\;\cos\;\alpha_{U,k}$, $D_{S}\;\mathrm{Sin}\;\alpha_{U,k}H_{S}$). The speed of the UAV, denoted as $v_{U}$, is characterized by its traveling direction, which may be represented by the angles γU,αandγU,β. The velocity of the user, denoted as $v_{k}$, is characterized by the directional angle γ_k,α. Therefore, it is necessary to revise the position for the $t_{th\;}$ time slot. Specifically, the coordinates of the AI may be expressed as $\left(0,0,H_{U}\;-\;v_{U}t\;sin\;\gamma U,\beta \right)$, while the coordinates of the $k_{th\;}$ user can be represented as (DkcosαU,k+vktcosγk,α,DksinαU,k+vktsinγk,α,0). Therefore, the revised values for the relative azimuth, elevation angles, and distances in equations (1), (2), (3), (4), (5), (6), (7) may be computed using the updated coordinates. Additionally, it was postulated that the UAV had an antenna with a constant physical flat position angle denoted as ψU. Likewise, the reflective surface of the IRS has a predetermined, physically flat position angle denoted as ψS. The distance between the antenna and the reflecting surface is denoted as Δ and is equal to half the wavelength (Δ=λ/2). Hence, the calculation of the propagation delay of the waves τk,m,τk,n,andτm,n may be performed accordingly.

The separations are evaluated as follows: the distance between the $m_{th}$ I element and the center ${\;O}_{U}$, indicated as Δm=(M+1−2m)Δ, and the distance in the nth element and the center O_S, denoted as Δn=1/2(N+1−2n)Δ. Equally, the distances encompassing the $k_{th}$ user and the mth on the AI antenna ($L_{k,m}$), the $k_{th}$ user and the nth on the IRS ($L_{k,n}$), and the $m_{th}$ element on the AI antenna and the n-th element on the IRS (L) are computed in accordance with equations (12), (13), (14).

### 3.1 Proposed channel modeling algorithm based on artificial intelligence

The utilization of a data-driven deep learning framework has emerged as a prominent strategy in the design and implementation of channel estimates. This trend is attributed to the innate capability of deep learning algorithms to extract intricate features from environmental information embedded in received signals, all without necessitating prior knowledge about channel statistics (Ma & Sun, 2020). Moreover, it's noteworthy to recognize that deep learning algorithms exhibit relatively modest computational complexity owing to their reliance on fundamental operations such as multiplications.

However, it is imperative to acknowledge a notable limitation of current deep learning-driven channel estimation approaches: the omission of the temporal dimension of CSI. Specifically, the incorporation of neighboring observations of the time-evolving CSI could potentially enhance prediction precision. To address this, efforts should be directed towards augmenting the deep learning models to encompass temporal correlation patterns in CSI dynamics.

**Fig. 2:**
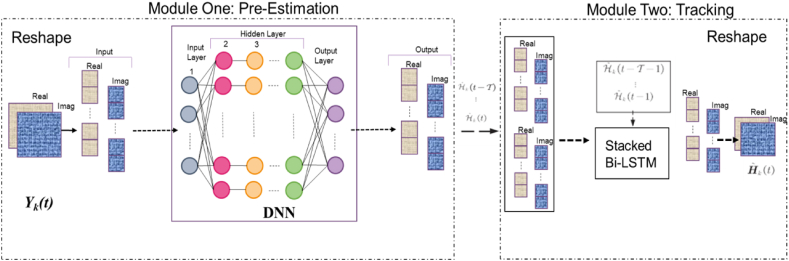
The illustration framework.

In order to effectively monitor dynamic channel conditions, it becomes imperative to equip neural networks with the capability to acquire knowledge about these temporal correlation patterns. This enhancement would not only improve the accuracy of channel estimation but also enable more robust and adaptive communication systems. There are two well-recognized approaches known as recurrent neural network (RNN) see Fig. 2.

Long Short-Term Memory (LSTM) networks have gained widespread adoption for tackling time-varying problems, encompassing various domains such as natural language processing. Both methodologies consider both past and current input data to make predictions. Recurrent Neural Networks (RNNs), designed with feedback loops, retain and propagate information over sequential data. However, RNNs encounter difficulties in learning long-term temporal relationships primarily due to the vanishing gradient issue. In contrast, the LSTM model integrates input and forget gates to bolster the retention of long-term dependencies while mitigating the problem of gradient flow [27].

Within this section, we introduce a framework that synergizes the benefits of Deep Neural Networks (DNN) and a multilayer perception mechanism for discerning intricacies within intricate environments. Moreover, we integrate LSTM with diverse input/output layers to facilitate the temporal passage of information. This framework, known as DNN and then by Bi-LSTM, is meticulously tailored for temporal tracking of CSI within communication systems involving UAVs and IRS. Fig. 2 visually delineates the comprehensive structure of the algorithm at hand. This configuration encompasses two principal modules: a DNN for reducing pre-estimation, relying on a pre-programmed offline model, and a Bi-LSTM for tracking, capitalizing on historical sequence insights. To express the channel tracking challenge in vector notation, we employ the symbols $y_{k}\left(t\right)=vec(Y_{k}\left(t\right)\text{CM}\left(N+1\right)\times 1$ and $h_{k}\left(t\right)=vec\;\left(H_{k}\left(t\right)\right)\in \text{CM}\left(N+1\right)\times 1$.

### 3.2 Module 1: DNNs channel description for estimation framework

The DNN is a variant of the artificial network that has numerous hidden layers positioned between the layers. In particular, it should be noted that within the neural network architecture, each layer has several neurons, the resulting output is derived from the weighted summation of these neurons, which are subject to the operation of a non-linear function. The Sigmoid function, denoted as $Sigmoid\left(x\right)=\frac{1}{1+{\mathrm{e}}^{-x}}$, and the Rectified Linear Unit (ReLU) function, denoted as fReLU(x)=max(0,x) are often used nonlinear activation functions in DNNs. The pre-estimation DNN ith $L_{D}-2$ hidden layers out of a total of $L_{D}$ layers. The $l_{th}$ hidden layer of the network is composed of $N_{e}$ neurons, where $2\leq l\leq L_{D}-1$ and $1\leq n_{e}\leq N_{e}$. The input vector $y_{k}\left(t\right)$ in the deep neural network (DNN) is represented as $[R(y_{k}\left(t\right);\;I\left(y_{k}\left(t\right)\right)]\varepsilon \;R\;\text{MN}P\times 2$, $y_{k}\left(t\right)$ as $R\left(y_{k}\left(t\right)=[Ry_{k,1}\left(t\right)\right),\ldots .\;R\left(y_{k},M,Np\left(t\right)\right)]$,and $I\left(y_{k}\left(t\right)=[Iy_{k,1}\left(t\right)\right),\ldots .\;I\left(y_{k},M,Np\left(t\right)\right)]$ respectively. The number of neurons in the input layer is equal to 2MNp. In a similar manner, the output vector ${\hat{\;H}}_{k}\left(t\right)$ of the DNN may be represented as $R({\hat{h}}_{k}\left(t\right);\;I({\hat{h}}_{k}\left(t\right)]\;\varepsilon \;R^{M\;\left(N+1\right)\times 2}$. The number of neurons in the output layer is determined by the equation 2M(N+1). The quantity denoted as 16MNp represents the total count of neurons present on each hidden layer.

**Table:** 

**Algorithm 1:** Training of Module 1: DNN-based Pre-estimation of Channel
**Input:** Training received Signal $y_{k}\left(1\right),\ldots ,y_{k}\left(T\right)\text{,}$ training true channel information ${\;H}_{k}\left(1\right),\ldots ,H_{k}\left(T\right)$. **Output:** Trained DNN for pre-estimation **Initialization:** Randomized initial weights θ. 1 Generate a set of Training sequences $y_{k}\left(1\right),\ldots ,y_{k}\left(T\right)\text{,}$ and ${\;H}_{k}\left(1\right),\ldots ,H_{k}\left(T\right)$ with selected SNRs and pilot overheads size $N_{P}$. 2 Design the DNN-based Pre-estimation Framework with $L_{D}$ layers and $N_{e}$ neurons in each hidden layer. Set the learning rate and batch size. 3 **While** not convergence **do**. 4 Update weights θ by minimizing loss function in (17). **End while.**

**Fig. 3:**
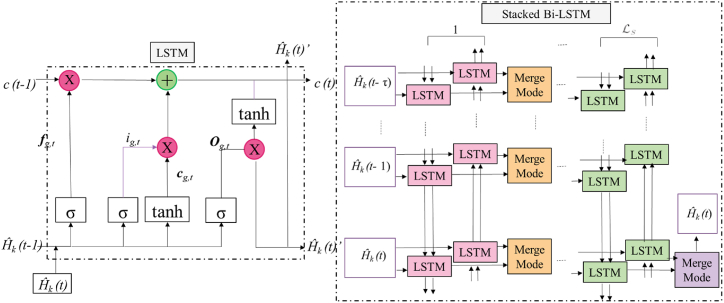
The structure of Bi-LSTM.

In order to articulate the transmission principle of DNNs, the notation $i_{th}$ is used to denote the input of neurons in the $l_{th}$ layer see Fig. 3. The variable $n_{e}^{th}$ denotes the output of the $n_{e}^{th}$ neuron at the $l_{th}$ layer. The weight matrix and bias vector of the $l_{th}$ layer are denoted as $W_{l}^{\left(DNN\right)}$ and ${\;b}_{l}^{\left(DNN\right)}$, respectively. Therefore, the output of each neuron may be represented as see in Eqs (12), (13), (14), (15).

(12) $$L_{k,m}=\sqrt{{\left(\left|Dk\;\sin\;\alpha \;U,k\right|-\;\Delta m\;\mathrm{Sin}\;\psi U\right)}^{2}+{\left(\left|Dkcos\alpha U,k\right|-\;\Delta m\;\cos\;\psi U\right)}^{2}z_{U}^{2},}$$

(13) $$L_{k,n}=\sqrt{{\left(\left|Ds,k\;\sin\;\alpha \;S,k\right|-\;\Delta n\;\mathrm{Sin}\;\psi s\right)}^{2}+{\left(\left|Ds,k\;\cos\;\alpha S,k\right|+\Delta n\;\cos\;\psi U\right)}^{2}z_{S}^{2},}$$

(14) $$L_{m,n}=\sqrt{{\left(\left|\text{Ds}\;\cos\;\alpha \;U,K\right|‐\Delta m\;\cos\;\psi s\right)}^{2}+{\Delta \text{ncos}\psi s)}^{2}+{\left(\left|\text{Dssin}\alpha \;U,S‐\Delta m\;\text{Sin}\psi U\right|\right)}^{2}+{\text{zU}‐z}_{S})}2$$

(15) $$ol,ne=fl,ne\left(b_{l,ne}^{\left(DNN\right)}+\omega^{\left(DNN\right)l,ne}\;T_{il}\right)\text{,}$$

The activation for the $l_{th}$ layer and the $n_{e}^{th}$ neuron is denoted as ${fl}_{n_{e}}$ During the training phase, when the total batch size is denoted as B, the output of the DNN with the $b_{th}$ orthogonal batch may be mathematically represented as Eq (16).

(16) $$Ĥk\left(\dot{b,}t\right)=f_{LD}\left(\ldots f_{2}\left(y_{k}\left(\dot{b,}t\right);\theta_{2}\right)\ldots ;\theta_{LD}\right)\text{.}$$

During the training phase for the development of a deep neural network (DNN), the parameter set $\theta_{l}=\left(w_{l}^{\left(DNN\right)},b_{l}^{\left(DNN\right)}\right)$ - representing the DNN model at the $l_{th}$ layer - can be obtained through the process of changing descent. This entails iteratively minimizing the loss function until result is attained. Formally, the loss function that spans all layers is defined as follows Eq (17):

(17) $$Loss\left(\Theta \right)=\frac{1}{2M\left(N+1\right)ß}\sum_{\dot{b,}}^{ß}({Ĥ}_{k\left(\dot{b,}t\right)-}H_{K}{\left(\dot{b,},t)\right)}^{2}\text{,}$$

Let *θ* denote the comprehensive collection of parameters across all levels, where $H_{k}(\dot{b,}$ $t)=\left[R\left(h_{k}\left(\dot{b,}t\right)\right);\;I\left(h_{k}\left(\dot{b,}t\right)\right)\right]$ indicates the actual value of channel in the $b_{th}$ batch. The procedural details for training of channel pre-calculation deep neural network (DNN) are outlined in Algorithm 1.

### 3.3 Module 2: channel tracking based on Bi-LSTM

The Bi-LSTM serves as the second component in the algorithm framework. It is responsible for monitoring the temporal sequence of Channel State Information (CSI) by using pre-estimated denoised channel information ${\hat{H}}_{k}\left(t-T\right)$ to ${\hat{\;H}}_{k}(t$) and the prior output of the channel tracking process ${\;\tilde{H}}_{k}\left(t-T\right)\;to\;{\tilde{H}}_{k}\left(t-1\right)$. In the model, the variable T represents the whole duration of the historical time slot that is used.

### 3.4 Long and Short-Term Memory (LSTM)

RNN, an extension of the recurrent neural network, is a learning algorithm that utilizes gradients to establish connections between past information and the present task [28]. Typically, temporal data is inputted into LSTM models in a sequential manner, following a forward trajectory through the chain-like architecture. This example specifically highlights the functioning of the first layer of the forward LSTM in the given illustration structure.

LSTM models offer several advantages over traditional RNNs. Firstly, LSTM models address the vanishing gradient problem inherent in RNNs, allowing them to effectively capture long-term dependencies in sequential data. This is achieved through the integration of input and forget gates, which enable the model to selectively retain or discard information over extended sequences. Additionally, LSTM models exhibit improved performance in tasks involving sequential data processing, such as natural language processing and time-series forecasting, due to their ability to preserve context information across multiple time steps.

However, despite their advantages, LSTM models also have limitations. One notable drawback is their increased computational complexity compared to simpler recurrent architectures like vanilla RNNs. This complexity arises from the additional gating mechanisms incorporated within the LSTM units, leading to higher training and inference times. Moreover, LSTM models may still struggle with capturing very long-term dependencies or contextual nuances in highly complex sequential data, particularly in scenarios where the data exhibits irregular or unpredictable patterns.

One notable distinction between LSTM and conventional neurals networks (CNN) is in the composition of hidden layers inside each LSTM cell. The first layer is referred to as the "forget layer," sometimes recognized as the $f_{g,t}$. The neural network architecture includes the incorporation of data from the preceding layer $\tilde{H}\left(t-1\right)$ and the current denoised input $\hat{H}\;\left(t\right)$ using weights ${\hat{\;w}}_{f}$, ${\hat{w}}_{f,}$ and bias ${\;b}_{f}$. Additionally, an activation function is applied to this combination of inputs in Eq (18).

(18) $$f_{g,t}=\sigma \;\left(\hat{w}\;f^{\hat{H}}k\;\left(t\right)+\tilde{w}f^{\tilde{H}}k\left(t-1\right)+b_{f}\right)\text{.}$$

The second layer can be written as in Eq (19).

(19) $$i_{g,t}=\sigma \;\left(\hat{w}\;i^{\hat{H}}k\;\left(t\right)+\tilde{w}i^{\tilde{H}}k\left(t-1\right)+b_{i}\right)\text{.}$$

The third layer is the cell input state $C_{g,t}$ that can be calculated as Eq in 20

(20) $$c_{g,t}=\tan\;h\;\left(\hat{w}\;c^{\hat{H}}k\;\left(t\right)+\tilde{w}c^{\tilde{H}}k\left(t-1\right)+b_{c}\right)\text{.}$$

final layer is called output gate ${\;O}_{g,t}$, which can be calculated as Eq in 21:

(21) $$o_{g,t}=\tan\;h\;\left(\hat{w}\;o^{\hat{H}}k\;\left(t\right)+\tilde{w}o^{\tilde{H}}k\left(t-1\right)+b_{o}\right)\text{.}$$

In summary, the construction of the hidden layers discussed above involves the use of matrix W and biasness vector b as respective frameworks. Symbol σ is often used to denote the activation function of a gate, typically implemented as a sigmoid function.

In addition to the cell $C_{g,t}$ inside the layers, the structure also includes two additional cell states: the previous output state c(t−1) which is sent to the LSTM cell, and the current cell output state c(t) which is transmitted to the subsequent LSTM cell. The status of the output at the present moment t may be modified in Eq (22).

(22) $$c\left(t\right)=f_{g,t}⊗\tanh\left(c\left(t\right)\right)\text{.}$$

Significantly, the traditional input layer refers to the better time sequence ${\hat{H}}_{k}\left(t\right)$ at slot t that is sent from DNN. Ultimately, the computation of the output layer may be determined as Eq 23

(23) $${\tilde{H}}_{k}{\left(t\right)}^{\prime }=\text{og},t⊗\tanh\left(c\left(t\right)\right)\text{.}$$

In order to address the limitation of a single LSTM cell, which is only capable of capturing historical information, researchers have introduced a bidirectional structure. This structure combines both the forward and backward directions, enabling the use of both past and future information. Consequently.

**Table:** 

**Algorithm 2:** Training of Module 2: Stacked Bi-LSTM-based Channel Tracking
**Input:** Pre-estimation ${\hat{H}}_{k}\left(1\right),\ldots ,{\hat{H}}_{k}\left(T\right)\text{,}$ training sequence, true channel information ${\;H}_{k}\left(1\right),\ldots ,H_{k}\left(T\right)$. **Output:** Trained model for tracking current sequence. **Initialization:** Randomized initial weights W and bias b. 1 Generate a new set of Training sequences ${\hat{H}}_{k}\left(1\right),\ldots ,{\hat{H}}_{k}\left(T\right)\text{,}$ and ${\;H}_{k}\left(1\right),\ldots ,H_{k}\left(T\right)$ with selected SNRs and pilot overheads size $N_{P}$ and T historic time step. 2 Design the bi-directional tracking Framework with $L_{S}$ stacked layers and T historic time step in each layer. Set the learning rate and batched size. 3 **While** not convergence do. 4 Update weights W and bias b by minimizing the loss function in (17). **End while.**

**Table:** 

**Algorithm 3:** Proposed Algorithm for overall Channel Tracking
**Input:** Received Signal $y_{k}\left(1\right),\ldots ,y_{k}\left(T\right)\text{.}$ **Output:** Channel tracking information $H_{k}\left(T\right)\text{.}$ **Initialization:** Randomized initial weights W and bias b. **Module 1 channel pre-estimation:** 1 Construct the $L_{D}$ layers DNN framework. 2 Load the DNN-optimized parameters that have been trained in Algorithm 1. 3 Pre-estimate the channel information ${\hat{H}}_{k}\left(1\right),\ldots ,{\hat{H}}_{k}\left(T\right)$. **Module 2 Tracking channel:** 1 Train the channel tracking model as in Algorithm 2 with (T−1) sequence data and T historic time step. 2 Use time sequence T−T to T−1 as input data to track T channel information ${\tilde{H}}_{k}\left(T\right)$ based on the trained tracking model.

The coming output ${\tilde{H}}_{k}{\left(t\right)}^{\prime }\left(f\right)$ and backward response ${\tilde{H}}_{k}{\left(t\right)}^{\prime }\left(f\right)$ of each cell are computed using the input relative to the output layer function as described in equation (23). The output of the forward layer is computed repeatedly using the time slots from t−Ttot−1. In a similar manner, the output of the backward layer is computed by using the reversed time sequence from t−1tot−T. The expression of a bidirectional structure may be stated as Eq (24).

(24) $${\tilde{H}}_{k}{\left(t\right)}^{\prime }=\sigma \;f,b({\tilde{H}}_{k}{\left(t\right)}^{\prime }\left(f\right)⊕{\tilde{H}}_{k}{\left(t\right)}^{\prime }\left(b\right)$$

Research has shown that the gradual improvement of performance may be achieved by using numerous hierarchical models in a stacked manner [29].

**Fig. 4:**
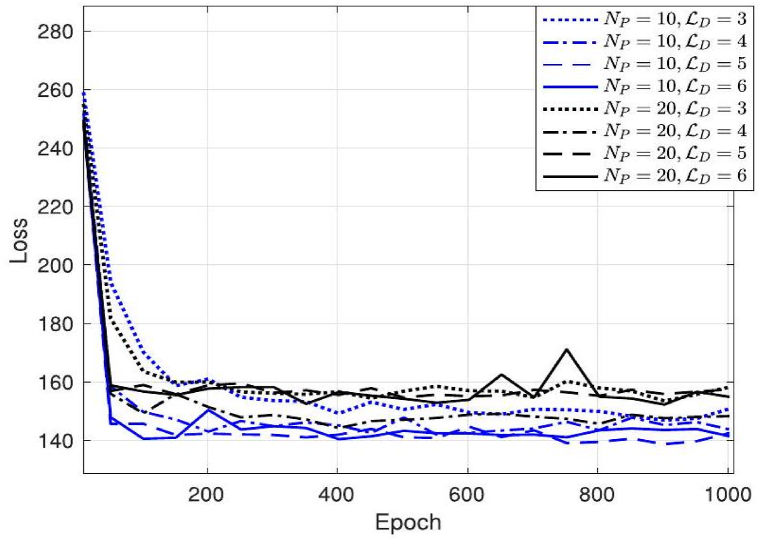
Illustrates the loss function for various sizes of pilot overheads.

Therefore, a stacked structure is used in which the output is afterward utilized as input to top layer, with the condition that the number of Bi-LSTM layers, denoted as ${\;L}_{s}$ be more than or equal to 2. The Bi-LSTM model incorporates bidirectional information and a deeper structure with T time slots in its process see Fig. 4. The resultant tracking output subsequent to the $L_{s}^{th}$ layer of Bi-LSTM may be expressed as Eq (25).

(25) $${\tilde{H}}_{k}\left(t\right)=\sigma \;f,b({\tilde{H}}_{k}{\left(t\right)}^{\prime }\left(f\right)Ls⊕{\tilde{H}}_{k}{\left(t\right)}^{\prime }\left(b\right)$$

denoted as $N_{p}$ ranging from 10 to 20. Additionally, the DNN is characterized by different total layers, denoted as ${\;L}_{D}$, which vary from 3 to 6.

The training procedure for the Stacked Bi-LSTM channel tracking model may be shown in Algorithm 2. The technique for tracking the whole channel is presented in Algorithm 3.

## 4 Results and discussion

In this section, we delve into the evaluation of the loss, channel tracking performance, and complexity of different methods within our established framework comprising two modules. Our analysis primarily centers on comparing the channel tracking performance of four models: DNN, DNN with LSTM, DNN with Bi-LSTM, and our recommended approach of using DNN followed by a Stacked Bi-LSTM model. The fundamental parameters are established in the following manner: The carrier frequency, denoted as ${\;f}_{c}$ is equal to 5.2 GHz. The speed of the UAV, represented as $v_{U}$ is 5 m/s. The azimuth angle of the AI speed, denoted as $r_{⋃,\propto }$ is π/6, while the elevation angle, represented as $r_{⋃,\beta }$ is also π/6. The speed of the $k_{th}$ user, denoted as ${\;v}_{k}$ is equal to 5 m/s, whereas the azimuth angle of the user's speed, represented as $r_{⋃,\propto }$ is π/24. In order to demonstrate the higher tracking capability of our suggested method, we conducted a simulation where we focused on a single user inside the system. The elements in the IRS system is denoted as N, and it is equal to 8. Similarly, the total number of antenna elements on the $k_{th}$ UAV is denoted as M, and it is also equal to 8. The angle of the antenna on the UAV is denoted as ${\;\psi }_{U}$ and is equal to π/6. Similarly, the surfaces on the IRS has a set flat angle denoted as ${\;\psi }_{U\;}$ and is also equal to π/6. The vertical altitude of the UAV, denoted as ${\;H}_{U}$, is measured to be 200 m. Similarly, the vertical altitude of the infrared sensor (IRS), denoted as ${\;H}_{S}$ is determined to be 100 m. IRS, and the $k_{th}$ user. The IRS is located at coordinates (−600 m, −600 m, 100 m), while the AI is positioned at (500 m, 600 m). The sequence consists of 200-time slots, with a train-test split rate of 0.8 used for evaluating the effectiveness of channel tracking.

**Fig. 5:**
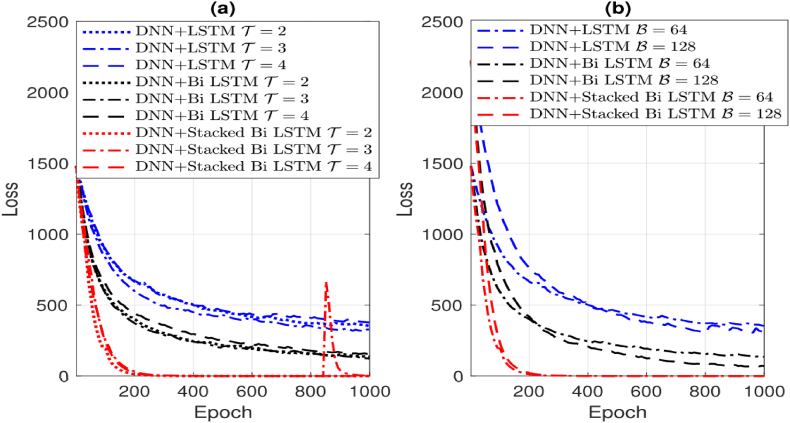
(a) presents the loss of several tracking algorithms with varying time steps. In this context, T represents a set of discrete time instances, namely T=2,3,4. The letter b denotes the batch size, which is a parameter that varies across different channel tracking techniques.

The loss function is a fundamental component in machine learning algorithms, particularly in the context of supervised learning. It quantifies the discrepancy between Fig. 4 illustrates the loss function of the channel pre-estimation DNN with various pilot overhead sizes $N_{p}$ and hidden layers ${\;N}_{D}$. The whole batch size is denoted as B see Fig. 5 (a) and (b).

The term "loss function" refers to a mathematical measure used to quantify the discrepancy between predicted and actual channel tracking outcomes. It is worth noting that the loss function may fluctuate across multiple channel tracking methods due to the influence of different batch sizes. The pre-estimation DNN is trained using a learning rate of 0.001 and a predefined value of 512 for ε. The whole training dataset is derived from a variety of signal-to-noise ratios (SNR) including −5, 0, 5, 10, 15, and 20 dB, as well as blockage probabilities $P_{k}$ of 0.3, 0.4, 0.5, and 0.6. The dataset consists of T=200 sequences. It is evident that when the learning rate $L_{D}$ is set to 3, both $N_{p}=10$ and $N_{p}=20$ need a greater number of epochs in order to get converged result. There is no difference seen in the convergence of function between $L_{D}$ values of 4, 5, and 6. The convergence loss with a pilot overhead size of $N_{p}=10$ is smaller compared to ${\;N}_{p}=20$. The rationale for this phenomenon is that when the number of processing units (NPs) rises, both the input and the number of neurals in every layer are correspondingly augmented, leading to a more intricate architecture. Based on empirical evidence, our approach demonstrates the ability to produce significant performance improvements with a very modest value of $N_{p}$. Therefore, the values $L_{D}=4$ and $N_{p}=10$ are chosen for the subsequent simulations.

In addition, Fig. 5 presents a comparison of the loss function across several channel tracking techniques, including LSTM, Bi-LSTM, and the suggested algorithm. The method under consideration exhibits a cumulative number of stacked layers denoted as $L_{s}$, which has a value of 3. The training channel sequence for difference tracking techniques is configured with a signal-to-noise ratio (SNR) of 20 dB and a probability value of ${\;P}_{k}=0.3$. The overall length of the sequence is T=200. Fig. 5 (a) presents a comparison of several historical time steps (T) including T=2,T=3, and T=4. The comparison is based on a fixed value of B=64 and ε=0.02. In comparison to the other two techniques, the proposed algorithm demonstrates a faster convergence towards a loss value close to zero for certain epochs of the dynamic channel sequence. It is important to highlight that the suitable hyper-parameter T can be chosen through various methods, contingent on factors like distinct values of $v_{U}$ and ${\;v}_{k}$. In Fig. 5 (b), a comparison is made between two batch sizes, namely 64 and 128, with respect to the values of T=2 and ε=0.02. The algorithm under consideration has comparable convergence performance.

**Fig. 6:**
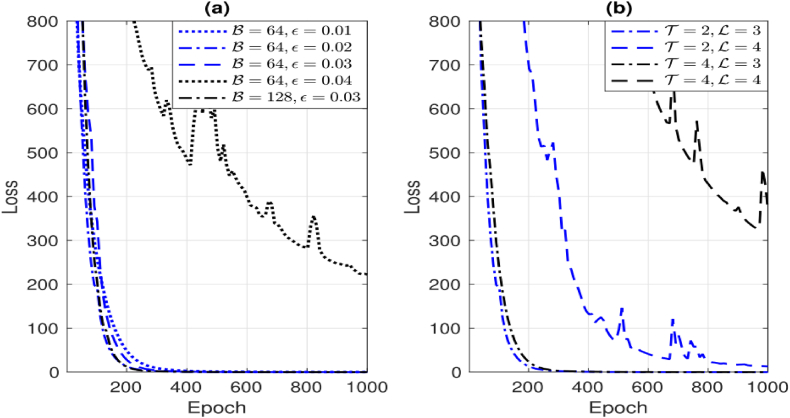
Illustrates the loss function behavior of the proposed algorithm under different batch sizes B and learning rates in (a), while (b) showcases the loss function variation with distinct historical time steps (T=2,4) and stacked layers $L_{s}=(3,4$).

**Fig. 7:**
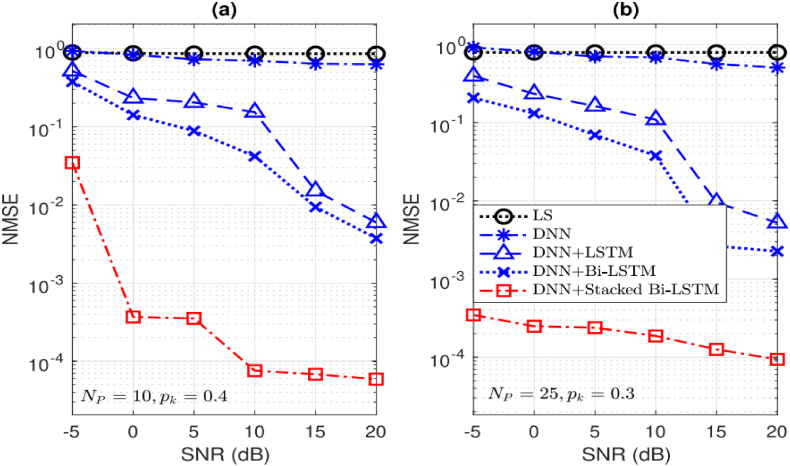
Fig. 7 displays the normalized mean square error (NMSE) performance of several techniques. (a) Np=10. (b) Np=25.

Furthermore, Fig. 6 illustrates the loss function of the method presented in this study with various hyper-parameters. The technique is designed for channel tracking in a dynamic channel scenario with a total duration of T=200. The SNR is established at 20 dB, while the pilot overhead size is indicated as ${\;N}_{P}=10$. For the subsequent evaluation of channel tracking performance, the hyper-parameter set that converges the loss function in a slightly lesser number of epochs is selected see Fig. 7 (a) and (b). This selection is made by comparing various learning rates in Fig. 6(a) and examining the values of T and $L_{s}$ in Fig. 6(b) with ε=0.01, B=64, T=2. and $L_{s}=3$.

### 4.1 Performance of channel tracking

The normalized mean square error (NMSE) has been selected as the statistic to assess the tracking performance of the algorithms [30] in Eq (26).

(26) $$NMSE=\frac{E(\|{\tilde{H}}_{k}-H_{k}{\|}^{2}\;}{E(\|H_{k}{\left\|\right)}^{2}}$$

The variable ${\tilde{H}}_{k}$ represents the estimated tracking channel, whereas $H_{k}$ denotes the genuine channel. In order to assess the performance of the algorithms in terms of NMSE, the whole sequence of T=200 is partitioned into two sets: a training set and a testing set. In our evaluation process, we divided the dataset into a training set comprising time slots 1 to 160, used for training the LSTM, Bi-LSTM, and our suggested algorithms. The testing set, encompassing time slots 161 to 200, was then utilized to assess tracking performance. This evaluation was conducted by calculating the average throughout a total of 500 rounds. Additionally, we employed a traditional least square (LS) method as a benchmark in our system.

Fig. 7 illustrates a noticeable reduction in the NMSE performance of all algorithms as the SNR increases. Notably, our investigated algorithm demonstrates significantly enhanced performance compared to alternative algorithms, even when pilot overheads are minimal. It's important to highlight that LS and DNN are stationary estimation techniques, analyzing incoming signals solely for estimation purposes. In contrast, the subsequent three algorithms exhibit improved performance by considering correlations across the temporal domain.

Our proposed approach showcases adeptness in extracting information from past and future contexts, as well as from intricate network structures, particularly through the utilization of bi-directional and layered architectures. This highlights the effectiveness of our method in leveraging temporal correlations and network complexity to achieve superior tracking performance see Table 2.

**Table 2:** Recorded operation time, measured in milliseconds, for both channel pre-estimation and tracking.

Module 1: Pre-Estimation DNN Layers				
M=9,N=9				M=8,N=4
	Np=11	Np=16	Np=21	Np=12
$\text{DNN}\;L_{D}=3$	0.210	0.342	0.328	0.067
$\text{DNN}\;L_{D}=4$	0.335	0.559	0.577	0.117
$\text{DNN}\;L_{D}=5$	0.422	0.713	0.724	0.144
$\text{DNN}\;L_{D}=6$	0.552	0.886	0.925	0.182

**Module 2: Channel Tracking Algorithms**				
M=9,N=9			M=4,N=8	
Np=11	T=3	T=6	T=3	T=6
LongShortTermMemory(LSTM)	2.957	2.986	0.288	0.394
Bi−LSTM	4.635	5.217	0.458	0.712
$\text{Stacked}\;\text{Bi}-\text{LSTM}\;L_{s}=2$	8.777	10.549	0.734	1.445
$\text{Stacked}\;\text{Bi}-\text{LSTM}\;L_{s}=3$	12.818	15.478	1.223	2.166

### 4.2 Complexity

This section presents an analysis of algorithmic complexity and computational time across different methods. The complexity of the pre-estimation DNN, excluding the bias term, can be represented as the summation of products of neuron counts in each layer, given as $Ꝺ\left(\sum_{l=1}^{LD-1}{(N}_{e}^{l}{\;N}_{e}^{l+1})\right)$ 1), where $N_{e}^{l}$ signifies the number of neurons in each layer. Specifically, the input layer (l=1) comprises 2MNp neurons, while each hidden layer ($l=2),\ldots .,\left(L_{D}-1\right))$ contains 16MNp neurons. The output layer $l=L_{D}$ consists of 2M(N+1) neurons. The computational complexity of a single LSTM cell can be calculated using the expression $Ꝺ\left(N_{C}\left(4N_{C}+4N_{2}+4N_{o}+3\right)\right)$ [31].

In this context, $N_{C},N_{i},N_{o}$ represent the quantities of memory cells, input units, and output units, respectively. It is worth noting that both ${\;N}_{i}\;and\;{\;N}_{o}$ are equal to 2M(N+1). Furthermore, in the case of LSTM, the number of cells is denoted as LSTM $N_{C}=1\tau$, while for Bi-LSTM, it is represented as ${\;N}_{C}=2\tau$. Similarly, for Stacked Bi-LSTM, the number of cells is denoted as ${\;N}_{C}=2\tau \;L_{s}$. The use of Bi-LSTM involves the estimate of both forward and backward directions, resulting in a total of ${\;N}_{C}=2\tau$. The equation ${\;N}_{C}=2\tau \;L_{s}$ holds true for Stacked Bi-LSTM, where $L_{s}$ is the number of layers of Bi-LSTM that are stacked together.

As indicated in Table 2, the runtime of the Python Jupyter Notebook escalates with the augmentation of parameters such as $N_{D}$ (number of layers), $N_{p}$ (number of parameters), and M (model complexity) for DNNs with multiple layers. This increase in runtime is expected due to the higher computational demands associated with deeper networks and increased model complexity.

Moreover, it's noteworthy that the Stacked Bi-LSTM model requires a longer computational time compared to other models due to its intricate architecture. Despite this longer runtime, the model demonstrates significantly improved performance in terms of NMSE when applied to dynamic channel sequences, as evidenced in the preceding paragraph.

In essence, while the Stacked Bi-LSTM model may necessitate more computational resources and time for processing, its enhanced performance in accurately modeling dynamic channel behaviors justifies the investment, highlighting its efficacy in capturing temporal dependencies and improving predictive accuracy.

## 5 Conclusion

In this study, the researchers developed a dynamic channel model in 3D geometry tailored for a communication system leveraging an Intelligent Reflecting Surface (IRS) to enhance UAV-enabled communication. They paid close attention to the intricate movement patterns of mobile users and navigation trajectories of UAVs when formulating the time-varying channel framework.

The channel model revolves around two primary connectivity components: a dynamic Line-of-Sight (LoS) link connecting users and UAVs, and a dynamic virtual LoS connection linking users and Infrared Small Unmanned Aerial Vehicles (IRSUAVs). Additionally, they introduced an innovative deep learning-driven methodology for channel tracking.

This methodology comprises two integrated modules: an initial channel estimation process using a DNN to mitigate noise effects, and a Stacked Bidirectional Long Short-Term Memory (Bi-LSTM) network dedicated to tracking the evolving channel. The architecture of the Stacked Bi-LSTM network is carefully designed with a bidirectional structure spanning multiple stacked layers, enabling effective capture and analysis of temporal information from past instances.

Simulation results indicate that the proposed approach for channel tracking outperforms several benchmarks while maintaining minimal pilot overheads and equal computing complexity. This suggests that the developed methodology holds promise for enhancing communication systems in UAV-enabled scenarios.

One limitation of this study is the reliance on simulation-based evaluations, which may not fully capture real-world complexities and variations. Additionally, the proposed deep learning-driven methodology for channel tracking may require extensive computational resources and training data, limiting its practical applicability in resource-constrained environments.

## Ethics approval and consent to participate

Not applicable.

## Consent for publication

All of the authors consented to publish this manuscript.

## Data availability

All relevant data in form of figures and results have been included in this paper. Corresponding author (qiang999xiaohu@163.com) may be contacted for any further query regarding data availability.

## CRediT authorship contribution statement

**Chongyang Liu:** Conceptualization, Formal analysis, Visualization, Writing – original draft, Writing – review & editing. **Xiaohu Qiang:** Conceptualization, Data curation, Formal analysis, Visualization, Writing – original draft, Writing – review & editing.

## Declaration of competing interest

The authors declare that they have no known competing financial interests or personal relationships that could have appeared to influence the work reported in this paper.

**Funding**

This article is supported by the Scientific Research Project of Hunan Provincial Department of Education: Exploration and Practice of the Construction Model of Science and Technology Innovation Platform in Higher Vocational Colleges from the Perspective of Industry Institute (Project Number: 22C1215).
